# Supplementary material for: Macrophage Stimulated by Low Ambient Temperature Hasten Tumor Growth via Glutamine Production
Source: Biomedicines. 2020 Sep 26;8(10):381. doi: 10.3390/biomedicines8100381 (PMC7600495; doi:10.3390/biomedicines8100381)
Supplement: Supplementary file 1 [file biomedicines-08-00381-s001.pdf]

Supplementary materials

Supplementary methods

Antibodies and reagents

Antibodies for glutamine synthetase (Glul), p21, cyclin A2, and cyclin E1 were purchased from Abcam (Cambridge, UK). Cyclin D1 and glyceraldehyde-3-phosphate dehydrogenase (GAPDH) were supplied by Santa Cruz Biotechnology (Santa Cruz, CA, USA). The anti-β-actin antibody, 3-isobutyl-1-methylxanthine (IBMX), insulin, dexamethasone, *L*-glutamine, and *L*-glutamic acid γ-(p-nitroanilide) hydrochloride (GPNA) were obtained from Sigma-Aldrich (St. Louis, MO, USA). Amicon® Ultra 2 mL centrifugal filters (3 K) were purchased from Merck Millipore (Burlington, MA, USA).

Cell viability assay

The viability of cancer cells treated with culture media (CM) from RAW264.7 cells, NIH/3T3 cells, differentiated 3T3-L1 cells, and bone marrow-derived macrophages (BMDM) cells was measured using a water-soluble 3-(4,5-dimethylthiazol-2-yl)-2,5-diphenyltetrazolium bromide (MTT) assay. RAW264.7 cells, NIH/3T3 cells, and BMDM cells (1×10^5 cells/58 cm^2) were cultured at 37°C or 33°C for 48 h. Cancer cells (5×10^3 cells/9.6 cm^2) were cultured at 37°C or 33°C for 72 h. MTT solution (2.0 mg/mL) was added into each well and the culture plates were incubated for 4 h. Thereafter, the CM was removed, and cells were lysed in a solubilization solution (EtOH: DMSO; 1:1). The amounts of purple formazan products in the colored solution were recorded by measuring the absorbance at 540 nm using a SpectraMax M2 microplate reader (Molecular Devices, Sunnyvale, CA, USA).

Differentiation of 3T3-L1 pre-adipocytes

Pre-adipocytes 3T3-L1 cells were fully cultured in the plates (58 cm^2) and maintained in DMEM containing 10% (v/v) BCS and antibiotics. After 2 d, post-confluent 3T3-L1 cells were stimulated with MDI induction media (MDI; 50 μM/mL IBMX, 1 μg/mL insulin and 1 μM/mL dexamethasone) in

DMEM containing 10% (v/v) FBS and antibiotics (100 U/mL penicillin and 100 μg/mL streptomycin) for 2 d. After 2 d, the induced differentiation of 3T3-L1 cells was subsequently changed with insulin media (1 μg/mL insulin) for 2 d. After 2 d, differentiated 3T3-L1 cells were changed with DMEM containing 10% (v/v) FBS and antibiotics every 2 d. On day 8, adipocyte differentiation was achieved and used for experiments.

Isolation and culture of bone marrow-derived macrophages

Bone marrow cells were isolated from the femur and tibia of male C57BL/6 mice and cultured in RPMI 1640 medium containing 10% (v/v) heat-inactivated FBS and antibiotics (100 U/mL penicillin and 100 μg/mL streptomycin) in the presence of 10 ng/mL murine M-CSF (Peprotech, Canton, MA, USA). After 4 d, non-adherent cells were collected and cultured for a further 3 d in 10 ng/mL murine M-CSF to derive BMDM. On day 7, adherent cells were harvested and used for experiments.

RAW264.7 and LLC cells co-culture

For co-culture experiments, RAW264.7 cells were cultured on the 0.4 μm pore size transwell insert (Corning Co., Corning, NY) and LLC cells were cultured in the bottom well of the transwell chamber. RAW264.7 cells (1×10^5 cells/4.52 cm^2) were cultured at 37°C or 33°C for 48 h. Cancer cells (5×10^3 cells/9.6 cm^2) were cultured at 37°C or 33°C for 72 h. Then, RAW264.7 cells in the upper insert were added on the top of each 6-well and cultured every day for 72 h.

Blood cell counts

Mice were sacrificed by carbon dioxide inhalation and blood was subsequently collected using Pasteur pipette by retro-orbital puncture. Collected blood was analyzed using a hematology analyzer (Hemavet 950FS, Drew Scientific, Miami Lakes, FL, USA).

Immunohistochemistry analysis

Immunohistochemistry was performed using DAKO Envision Polymer techniques (Agilent technologies, CA, USA). Briefly, formalin-fixed, paraffin-embedded tissue sections of 3 μM thickness were deparaffinized and rehydrated. The tumor tissues were 10% formalin-fixed, paraffin-embedded tissue sections of 3 μm thickness were deparaffinized and rehydrated. Heat-induced epitope retrieval was performed using citrate buffer (pH 6.0) in pressure cooked (IHC World, LLC., a steamer set 120 v, 60 Hz, 650 w) for 45 minutes. The endogenous peroxidase activity was blocked using 3% H_2O_2 in methanol for 10 minutes. Tissue sections were then incubated for 60 min with mouse monoclonal anti-CD68 antibody (1:100; Santa Cruz Biotechnology, Santa Cruz, CA, USA), at room temperature in a humidified chamber. After washing 3 times for 5 min with TBS, tissue slides were incubated with EnVision+ System- HRP Labelled Polymer Anti-Mouse as per the manufacturer's instructions (DAKO). Tissue sections were incubated with 3,3'-diaminobenzidine/H_2O_2 (DAKO) for color development and counter-stained with Mayer's hematoxylin. The stained tissue slides were dehydration and mounting in synthetic mountant (Thermo Fisher Scientific, Rockford, IL, USA). Microscopic Images took an OLYMPUS BX53 and DP70 (Olympus, Tokyo, Japan) microscopic digital camera and Images were analyzed using LEOPARD v.2.0 software (ZOOTOS, Uiwang, Korea).

Glutamine analysis by HPLC

The glutamine content of CM and whole-cell from RAW264.7 cells, NIH/3T3 cells, and differentiated 3T3-L1 cells were determined using high-performance liquid chromatography (HPLC). In addition, the glutamine content of serum and tumor tissue removed from euthanized mice was determined using HPLC. These samples for glutamine analysis were prepared as previously described, with modifications [1,2]. Briefly, the cells cultured in growth medium at 37°C or 33°C for 48 h in biological triplicate. The cells and tumor tissue were then harvested using 80% MeOH and lysed by vigorous vortexing, completely. The supernatants were collected and centrifuged at 21,130 ×g and

4°C for 10 min. The aqueous phase was mixed with chloroform by liquid-liquid extraction. The supernatants were collected and dried using a vacuum centrifuge. The sample was reconstituted with 50% MeOH and subjected to HPLC analysis. A sample was injected using a 1260 series auto-sampler (Hewlett Packard, Palo Alto, CA, USA) with a C18 Nova-Pak 4 μm column (Waters, Milford, DE, USA). The mobile phase was initially gradient flow using 100% solvent A (140 mM NaHAc, 0.15% TEA, 0.03% EDTA, 6% CH_3CN, pH 6.1) to 100% solvent B (60% CH_3CN, 0.015% EDTA) for 30 min. The flow rate was set at 1 mL/min, and glutamine was determined based on the measurement of the absorbance at 254 nm using an 1100 series UV spectrophotometer (Hewlett Packard).

Western blot analysis

The cells were harvested, lysed with 1% NP-40 lysis buffer (150 mM NaCl, 10 mM HEPES buffer (pH 7.4), 1% NP-40, 5 mM NaPyrophosphate, 5 mM NaF, 2 mM Na_3VO_4), containing 1% protease inhibitor cocktail (Roche, Mannheim, Germany) and centrifuged at 21,130 ×g for 10 min at 4°C. Protein concentration was determined by bicinchoninic acid (BCA) method, using bovine serum albumin (BSA) as standard. Equal amounts (20 μg) of proteins were separated on sodium dodecyl sulfate-polyacrylamide gel (SDS-PAGE) and transferred onto nitrocellulose blotting membrane (Hybond ECL; GE Healthcare, Pittsburgh, PA, USA). The membranes were blocked with 5% defatted milk (Sigma-Aldrich, St. Louis, MO, USA) in Tris-buffered saline containing 0.1% Tween-20 at room temperature for 1 h and were incubated with specific primary antibodies against each target protein overnight at 4°C. The membranes were washed thrice and incubated with appropriate secondary antibodies conjugated with horseradish peroxidase and detected using ECL chemiluminescence (Thermo Fisher Scientific, Rockford, IL, USA) using ImageQuant LAS 4000 (GE Healthcare).

Gene knockdown with small interfering RNA (siRNA)

RAW264.7 cells (5×10^5 cells/58 cm^2) were transfected with siRNA at a final concentration of 50

nM using Lipofectamine® RNAiMAX Reagent (Thermo Fisher Scientific) for 24 h and cultured at 37°C or 33°C for 48 h. The knockdown efficiency of siRNA was verified by qRT-PCR and western blot analysis. The sequence of the Glul siRNA-1 was 5′-GGTTGATGGTACCGGAGAA-3′, Glul siRNA-2 was 5′-CAACGAAACAGGCGACGAA-3′ and the control (non-specific) siRNA was obtained from Bioneer (Daejon, Korea).

qRT-PCR analysis

Total RNA of the cells was extracted using Trizol reagent (GeneAll, Lisbon, Portugal), and cDNA was synthesized using a RevertAid First Strand cDNA Synthesis Kit (Thermo Fisher Scientific). Quantitative real-time polymerase chain reaction (qRT-PCR) was performed using the QuantiNova™ SYBR®Green PCR kit (QIAGEN) and a Qiagen Rotor-Gene® Q Real-Time PCR Detection System according to the manufacturer's instructions. The relative expression level of mRNAs was calculated by the comparative threshold cycle method using 36B4 as an internal control. The following primers were used in these studies: *Gls* forward 5′-ATGCATTCCCCAGCCTGATG-3′, reverse 5′-GGTGCTCAATTACCTGAATCACA-3′; *Glul* forward 5′-AGTCTGAAGGCTCCAACAGC-3′, reverse 5′-TCCATTCCAAACCAGGGGTG-3′; *Slc7a5* forward 5′- CTGGTCTTCGCCACCTACTT-3′, reverse 5′- GCCTTTACGCTGTAGCAGTTC-3′; *Slc7a8* forward 5′- AAGAAGCCTGACATTCCCCG-3′, reverse 5′- TGTGTTGCCAGTAGACACCC-3′; *Slc1a5* forward 5′-CCCCTCCTGAAACAGTACCA-3′, reverse 5′-AGCCTCTCCAGGAAGGAGAC-3′; *36B4* forward 5′-AACTTTGGCATTGTGGAAGG-3′, reverse 5′-ACACATTGGGGGTAGGAACA-3′.

BrdU incorporation assay

Proliferation in LLC cells was measured by the BrdU cell proliferation assay kit (BioVision, Milpitas, CA, USA). Briefly, LLC cells (5×10^3 cells/0.32 cm^2) treated with RAW264.7 CM (1×10^5 cells/58 cm^2) for 24 h. BrdU solution was added to the wells and incubated at 37°C for 4 h in an atmosphere containing 5% CO_2. The culture medium was removed, 100 μL fixing/denaturing solution was added to each well and incubated at room temperature for 30 min. The supernatant was removed, 100 μL 1X BrdU detection antibody solution was added to each well and incubated at room

temperature. After 1 h, wells were washed with 1X wash buffer and incubated with 100 μL 1X anti-mouse HRP-linked antibody solution to each well at room temperature. After 1 h, wells were washed with 1X wash buffer and 3,3′, 5,5′-tetramethylbenzidine (TMB) substrate (100 μL) was added into each well and then absorbance was measured at 650 nm for 30 min. To terminate color development, 100 μL stop solution was added into each well and wells were measured at 450 nm.

Flow cytometry analysis

For serum starvation, LLC cells (5×10^4 cells/58 cm^2) were cultured in DMEM without serum for 48 h and re-stimulated with RAW264.7 CM (3×10^5 cells/58 cm^2) for 24 h. The cells were collected and centrifuged at 2,500 ×g and 4°C for 10 min. The cells were washed twice in PBS buffer. The cells were gently resuspended in 100 μL PBS and fixed with the serial addition of 200 μL PBS containing 10% ethanol/5% glycerol and 200 μL PBS containing 50% ethanol/5% glycerol. The samples were incubated on ice for 5 min and then added to 1 mL of PBS containing 70% ethanol/5% glycerol in the final. The cells were kept at 4°C overnight, washed with PBS and centrifuged at 2,500 ×g for 10 min at 4°C. The samples were resuspended with 0.5 mL of propidium iodide (PI) solution (40 μg/mL PI, 100 μg/mL RNase, 0.1% Triton-X 100) at 37°C for 30 min. The relative DNA contents of the stained cells were analyzed using a flow cytometer (BD FACS Canto II, BD Biosciences, San Jose, CA, USA) with an excitation/emission wavelength of 495/605 nm.

Supplementary References

1. Seo, J.-W.; Choi, J.; Lee, S.-Y.; Sung, S.; Yoo, H.J.; Kang, M.-J.; Cheong, H.; Son, J. Autophagy is required for PDAC glutamine metabolism. *Scientific Reports* **2016**, *6*, 37594, doi:10.1038/srep37594

2. Kato, S.; Masuda, Y.; Konishi, M.; Oikawa, T. Enantioselective analysis of D- and L-amino acids from mouse macrophages using high performance liquid chromatography. *Journal of*

pharmaceutical and biomedical analysis **2015**, *116*, 101-104, doi:10.1016/j.jpba.2015.04.028.

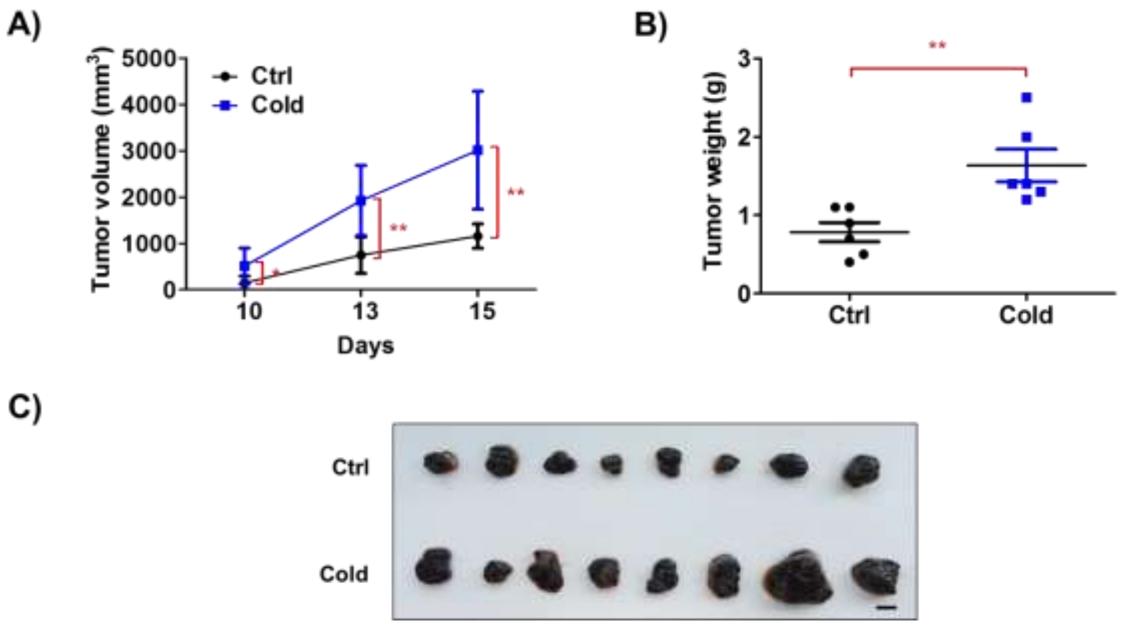

Figure S1. Ambient cold accelerated the growth of allograft melanoma B16F10 cells. B16F10 cells (1 × 10^6 cells/ 100 μL PBS) were subcutaneously injected into the dorsum of mice. (a, b) After 15 d, the tumor volume and weight were measured. (c) Images of tumor samples from control and ambient cold groups are shown. A representative bar (1 cm) was demonstrated. Data are expressed as the mean ± SD. **p* < 0.05 and ***p* < 0.01 compared to the control group (22°C).

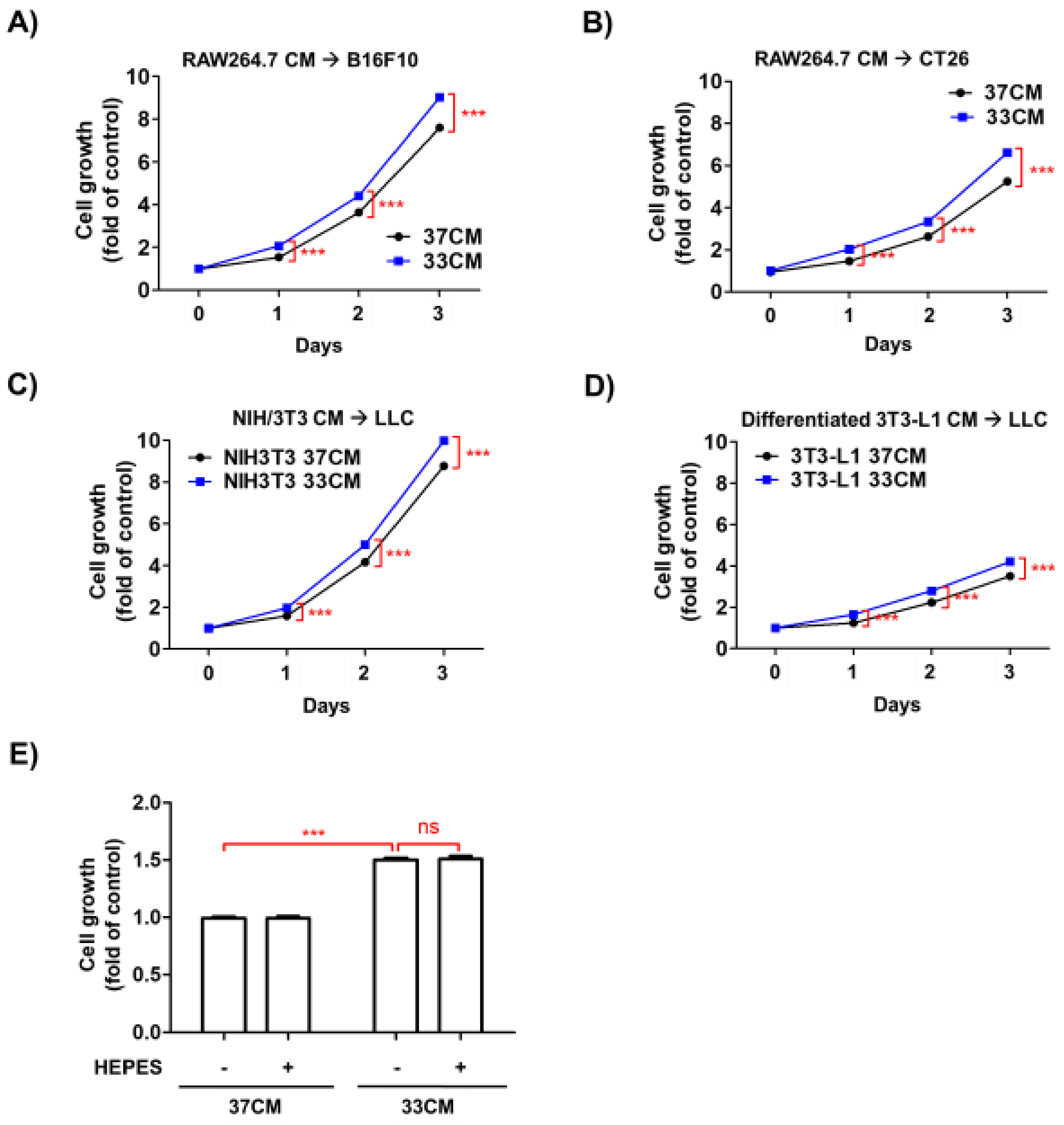

Figure S2. The growth of cancer cells were enhanced by cold-exposed stromal cells. (a-d) The culture media from stromal cells incubated at 33°C or 37°C for 48 h were transferred to cancer cells. The cancer cells incubated at 37°C for 72 h. The growth of B16F10 cells treated with RAW264.7 cells (a) and CT26 cells incubated with RAW264.7 cells (b) were estimated by MTT assay. The growth of LLC cells incubated with NIH/3T3 CM (c) and differentiated 3T3-L1 CM (d) were measured by MTT assay. (e) CM from RAW264.7 cells was titrated to pH 7.4 with HEPES buffer. Data are expressed as the mean ± SD. ****p* < 0.001 compared to the control (37CM).

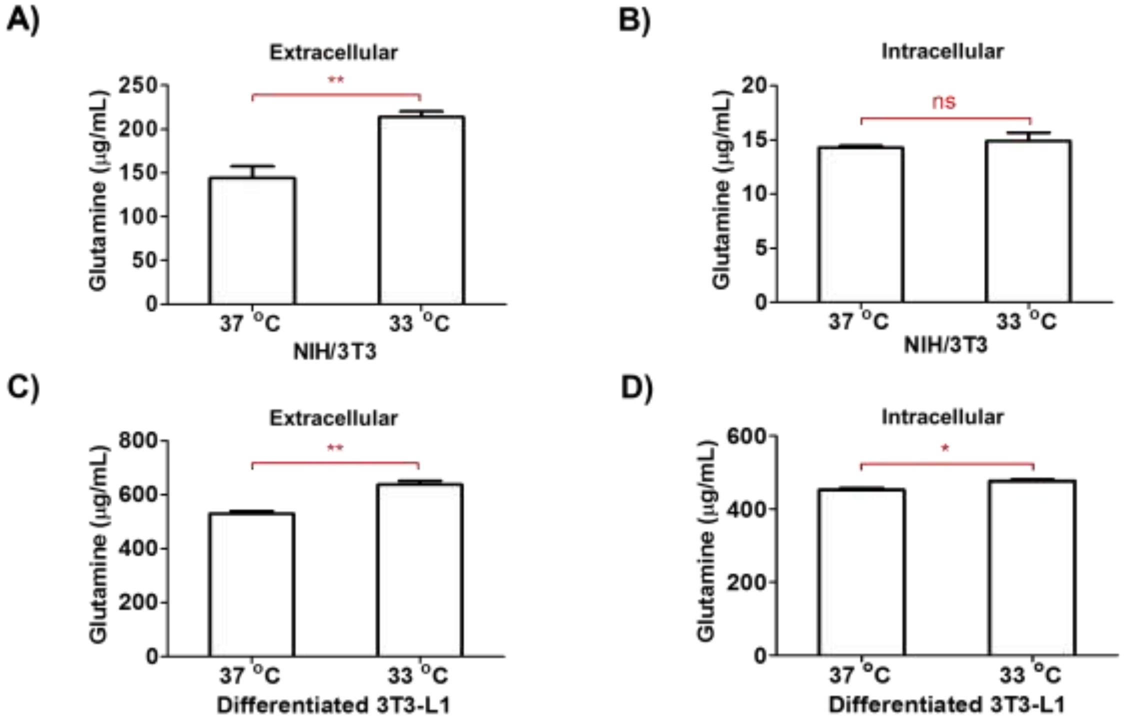

Figure S3. Glutamine contents in cold-exposed stromal cells. (a, b) NIH/3T3 cells were incubated at 33°C or 37°C for 48 h. (c, d) Differentiated 3T3-L1 cells were incubated at 33°C or 37°C for 48 h. The glutamine contents in CM (a, c) and in cell lysate (b, d) were measured using HPLC analysis. Data are expressed as the mean ± SD. *$p < 0.05$ and **$p < 0.01$ compared to the control (37°C).

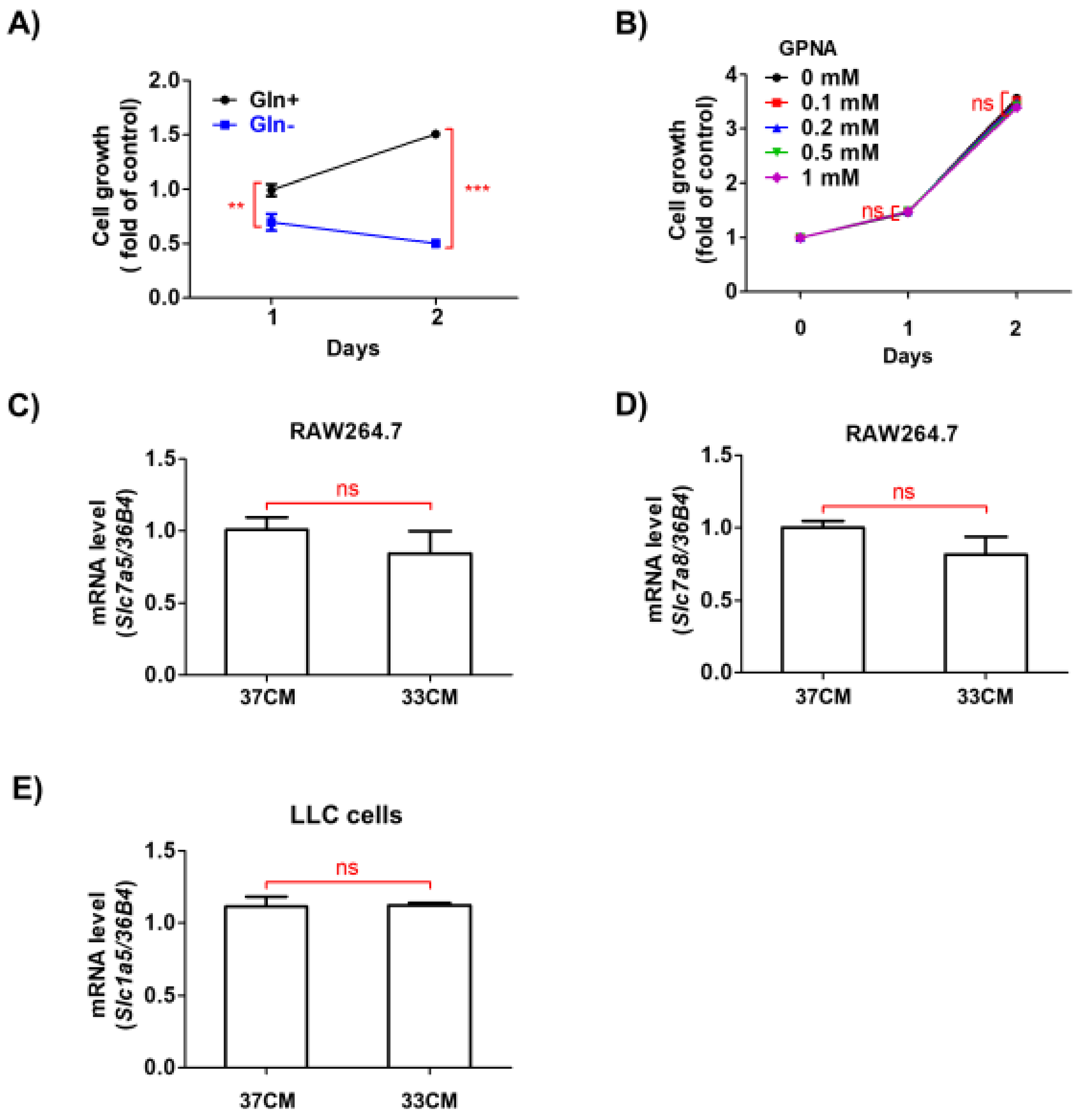

Figure S4. The effect of glutamine or GPNA on the growth of cancer cells. (a) LLC cells were incubated in complete media (Gln+, corresponding to glutamine 584 μg/mL) or glutamine-free media (Gln-) at 37°C for 48 h. The growth of LLC cells was examined using an MTT assay. (b) LLC cells were treated with the indicated concentration of GPNA for 48 h and the growth of LLC cells were measured by MTT assay. (c, d) The RAW264.7 cells were cultured at 33°C or 37°C and incubated for 48 h. The expressions of *Slc7a5* and *Slc7a8* were measured by qRT-PCR. The *36B4* was used as internal control. (e) The LLC cells were treated with RAW264.7 CM cultured at 33°C or 37°C and incubated for 24 h. The mRNA expression of *Slc1a5* was determined by qRT-PCR. The *36B4* was used as internal

control. Data are expressed as the mean ± SD. **$p < 0.01$ and ***$p < 0.001$ compared to the control. ns mean no significance.

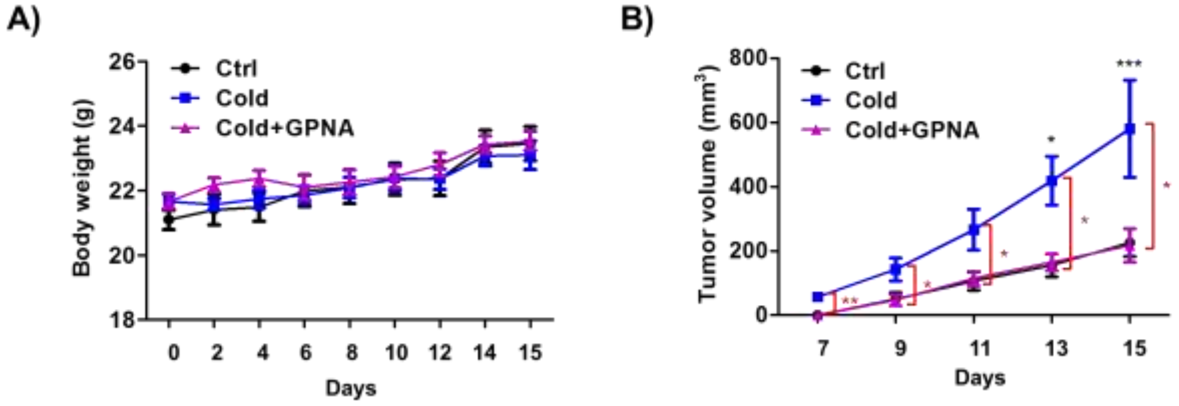

Figure S5. The effect of GPNA on the body weight and tumor growth in ambient cold conditions. LLC cells (1 × 10^6 cells/100 μL PBS) were subcutaneously injected into the dorsum of mice. GPNA (20 mg/kg) was intraperitoneally injected into the mice every day for 15 d. (a) Body weight (g) of each group was measured every 2 d. (b) The volume of tumor was measured every 2 d from 7 d after inoculation. Data are expressed as the mean ± SD. *$p < 0.05$ and **$p < 0.01$ compared to the control group (22°C).

A)

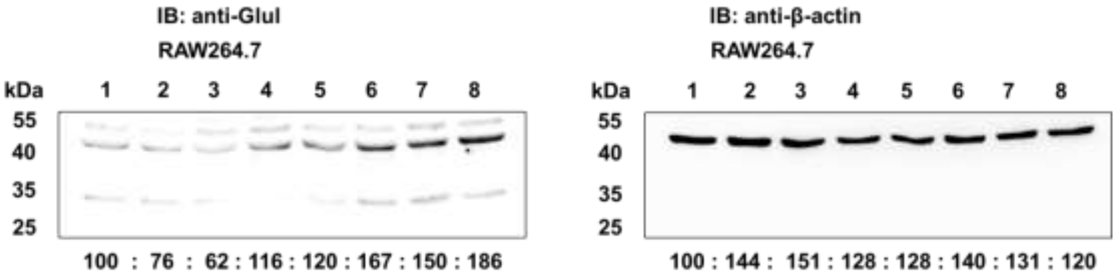
IB: anti-Glul
RAW264.7
kDa
1 2 3 4 5 6 7 8
55
40
35
25
100 : 76 : 62 : 116 : 120 : 167 : 150 : 186
IB: anti-β-actin
RAW264.7
kDa
1 2 3 4 5 6 7 8
55
40
35
25
100 : 144 : 151 : 128 : 128 : 140 : 131 : 120

B)

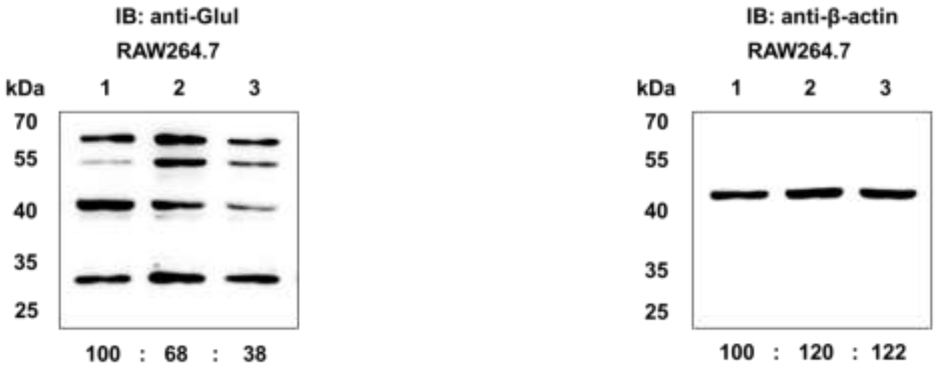
IB: anti-Glul
RAW264.7
kDa
1 2 3
70
55
40
35
25
100 : 68 : 38
IB: anti-β-actin
RAW264.7
kDa
1 2 3
70
55
40
35
25
100 : 120 : 122

C)

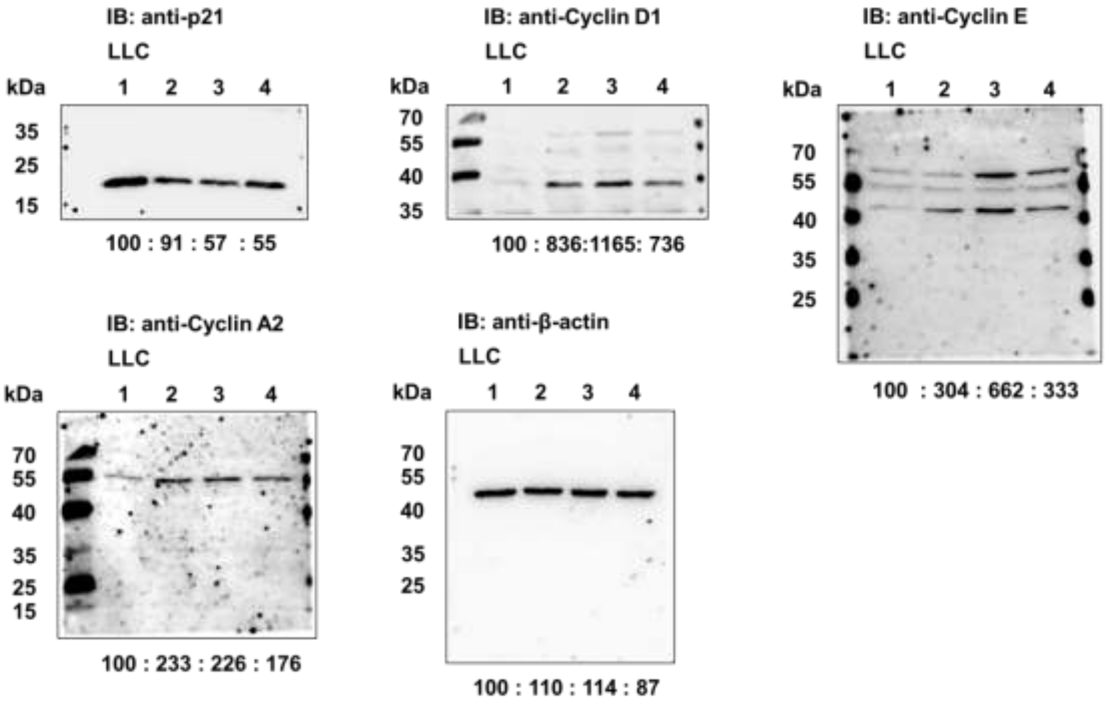
IB: anti-p21
LLC
kDa
1 2 3 4
35
25
15
100 : 91 : 57 : 55
IB: anti-Cyclin D1
LLC
kDa
1 2 3 4
70
55
40
35
100 : 836:1165: 736
IB: anti-Cyclin E
LLC
kDa
1 2 3 4
70
55
40
35
25
100 : 304 : 662 : 333
IB: anti-Cyclin A2
LLC
kDa
1 2 3 4
70
55
40
35
25
15
100 : 233 : 226 : 176
IB: anti-β-actin
LLC
kDa
1 2 3 4
70
55
40
35
25
100 : 110 : 114 : 87

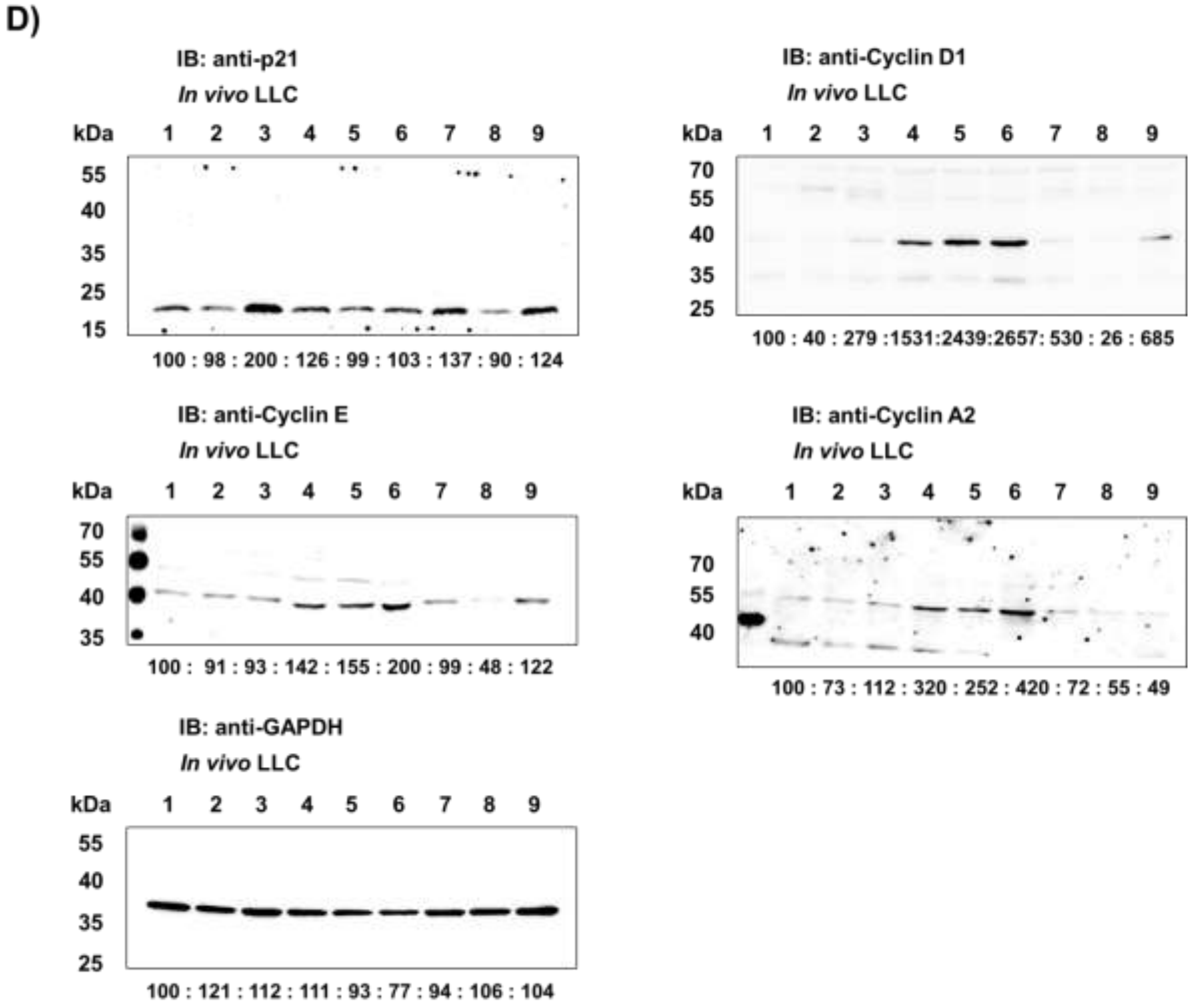

Figure S6. The whole blot used in this study. The figure shows the whole blot of (a) Figure 5b, (b) Figure 5d, (c) Figure 6e, (d) Figure 7f.

Table S1. Potential metabolites in cold-exposed macrophages cultured media found by CE-TOFMS.

	Relative area		Comparative ratio[a]
Compound name	37°C	33°C	33°C vs 37°C
1-Methyladenosine	2.2E-05	5.9E-06	0.3
1-Methylhistidine	3.6E-04	3.3E-04	0.9
1-Pyrroline 5-carboxylic acid	N.D.	2.9E-05	1<
2'-Deoxycytidine	1.2E-05	1.1E-04	9.0
2'-Deoxyuridine	7.3E-05	N.D.	<1
2-Aminobutyric acid	3.3E-04	3.9E-04	1.2
2-Hydroxybutyric acid	3.9E-05	3.2E-05	0.8
2-Hydroxyvaleric acid	7.5E-05	4.7E-05	0.6
2-Oxoglutaric acid	2.7E-04	1.4E-04	0.5
2-Oxoisovaleric acid	3.2E-04	3.1E-04	1.0
3-Hydroxybutyric acid	3.4E-04	1.5E-04	0.5
4-Acetamidobutanoic acid	1.6E-05	1.7E-05	1.1
4-Methyl-2-oxovaleric acid	1.6E-03	2.2E-03	1.4
5-Aminoimidazole-4-carboxamide ribotide	1.6E-05	N.D.	<1
5-Hydroxylysine	4.3E-05	3.2E-05	0.7
5-Oxoproline	1.4E-02	1.3E-02	1.0
7-Methylguanine	2.1E-05	N.D.	<1
N-Dimethylarginine	1.2E-04	6.4E-05	0.5
Alanine	2.2E-02	1.5E-02	0.7

Allantoic acid	2.9E-05	2.2E-05	0.8
Aminoacetone	2.7E-04	3.5E-04	1.3
Arginine	8.5E-03	1.7E-02	2.0
Argininosuccinic acid	1.1E-05	7.2E-06	0.7
Asparagine	5.7E-05	2.0E-04	3.4
Aspartate	6.5E-04	4.4E-04	0.7
Betaine	7.4E-04	1.2E-03	1.6
Carnitine	2.1E-04	2.5E-04	1.2
Carnosine	8.3E-05	8.6E-05	1.0
Choline	2.1E-04	3.1E-03	15
Citric acid	8.1E-03	5.1E-03	0.6
Citrulline	3.7E-04	3.7E-04	1.0
Creatine	1.9E-03	1.7E-03	0.9
Creatinine	1.3E-03	1.2E-03	0.9
Cystathionine	3.4E-05	3.6E-05	1.1
Cysteine glutathione disulfide	3.3E-04	2.2E-04	0.7
Cystine	4.3E-03	5.3E-03	1.2
Diethanolamine	2.0E-05	4.1E-05	2.1
Dihydroxyacetone phosphate	3.5E-05	N.D.	<1
Dyphylline	6.9E-04	1.8E-03	2.6
Folic acid	4.5E-05	5.1E-05	1.1
Fumaric acid	6.9E-05	3.6E-05	0.5
γ-Aminobutyric acid	N.D.	3.6E-05	1<

Glutamine	1.2E-03	5.7E-02	47
Glutamate	8.6E-03	6.6E-03	0.8
Gluconic acid	4.1E-05	3.8E-05	0.9
Gluconolactone	3.7E-05	2.6E-05	0.7
Glutathione disulfide	5.0E-05	1.8E-05	0.4
Glycine	6.6E-03	1.1E-02	1.7
Glyceric acid	N.D.	2.5E-05	1<
Glycerol	1.6E-03	4.8E-03	3.0
Glycerol 3-phosphate	1.4E-05	N.D.	<1
Glycerophosphocholine	1.5E-04	9.4E-05	0.6
Guanidoacetic acid	6.2E-04	1.9E-04	0.3
Hexanoic acid	2.1E-05	N.D.	<1
Hippuric acid	5.4E-05	6.0E-05	1.1
Histidine	6.7E-03	9.0E-03	1.3
Homocitrulline	1.9E-05	N.D.	<1
Homocystine	5.7E-05	1.4E-05	0.2
Homovanillic acid	2.0E-05	1.2E-05	0.6
Hydroxyproline	5.4E-04	5.6E-04	1.0
Isoleucine	3.9E-02	5.3E-02	1.4
Isethionic acid	1.8E-05	2.3E-05	1.3
Butyric acid	1.4E-04	4.1E-05	0.3
Isobutyrylcarnitine	4.1E-05	4.6E-05	1.1
Isocitric acid	3.0E-04	1.1E-04	0.4

Valeric acid	2.0E-04	4.7E-05	0.2
Kynurenine	1.7E-05	1.8E-05	1.1
Lactic acid	2.3E-01	8.5E-02	0.4
Leucine	3.7E-02	5.7E-02	1.5
Lysine	2.7E-02	3.4E-02	1.3
Malic acid	7.1E-04	3.6E-04	0.5
Methionine	4.2E-03	6.3E-03	1.5
Methionine sulfoxide	1.3E-04	1.8E-04	1.4
Mucic acid	1.8E-05	1.9E-05	1.0
N, *N*-Dimethylglycine	5.6E-05	6.5E-05	1.2
N-Acetylalanine	2.1E-05	N.D.	<1
N-Acetylneuraminic acid	2.7E-05	2.8E-05	1.0
N-Ethylglycine	4.9E-05	N.D.	<1
N^5-Ethylglutamine	9.7E-05	1.1E-04	1.1
N^6-Methyllysine	1.9E-04	1.9E-04	1.0
N^8-Acetylspermidine	2.3E-05	N.D.	<1
Nicotinamide	1.3E-03	1.3E-03	1.0
O-Acetylcarnitine	8.6E-05	9.8E-05	1.2
O-Acetylhomoserine	6.7E-05	6.6E-05	1.0
Octopamine	1.9E-04	1.6E-04	0.9
Ophthalmic acid	3.9E-05	N.D.	<1
Ornithine	3.4E-03	2.2E-03	0.7
Pantothenic acid	3.3E-04	3.1E-04	1.0

Phenylalanine	2.6E-02	3.2E-02	1.2
Phenaceturic acid	1.5E-05	1.8E-05	1.2
Phosphoenolpyruvic acid	3.2E-05	N.D.	<1
Phosphorylcholine	5.0E-05	2.3E-05	0.5
Proline	7.3E-03	3.3E-03	0.5
Propionic acid	2.5E-04	N.D.	<1
Pyridoxal	6.9E-05	6.6E-05	1.0
Pyridoxine	1.9E-03	2.1E-03	1.1
Pyruvic acid	3.0E-03	2.3E-03	0.8
S-Sulfocysteine	1.7E-05	2.3E-05	1.3
Symmetric dimethylarginine	4.4E-05	4.2E-05	0.9
Serine	2.4E-04	2.4E-03	10
Succinic acid	9.0E-04	8.5E-04	0.9
Sulfotyrosine	1.7E-05	1.5E-05	0.9
Terephthalic acid	4.5E-05	4.5E-05	1.0
Thiamine	9.8E-04	9.3E-04	0.9
Thiaproline	6.5E-05	N.D.	<1
Threonine	2.1E-02	2.6E-02	1.3
Threonic acid	1.9E-04	1.8E-04	1.0
Thymidine	5.6E-05	4.8E-05	0.9
Trimethylamine *N*-oxide	6.9E-05	7.3E-05	1.1
Tryptophan	2.0E-03	2.8E-03	1.4
Tyrosine	1.5E-02	1.8E-02	1.2

Urea	1.1E-02	1.1E-02	1.0
Uric acid	4.7E-05	3.7E-05	0.8
Uridine	1.1E-04	5.3E-05	0.5
Urocanic acid	1.9E-05	N.D.	<1
Valine	3.5E-02	4.4E-02	1.3
cis-Aconitic acid	3.2E-04	1.3E-04	0.4
β-Alanine	N.D.	3.2E-05	1<
γ-Butyrobetaine	2.8E-05	3.6E-05	1.3

N.D. (Not Detected): The target peak or metabolite was below detection limits.

[a]The ratio of comparative analysis is computed by using averaged detection values. The relative peak data by 33°C-cultured RAW264.7 CM was used as a molecule and peak data by 37°C-cultured RAW264.7 CM was used as the denominator.
